# Reduced oxidized LDL in T2D plaques is associated with a greater statin usage but not with future cardiovascular events

**DOI:** 10.1186/s12933-020-01189-z

**Published:** 2020-12-14

**Authors:** Pratibha Singh, Isabel Goncalves, Christoffer Tengryd, Mihaela Nitulescu, Ana F. Persson, Fong To, Eva Bengtsson, Petr Volkov, Marju Orho-Melander, Jan Nilsson, Andreas Edsfeldt

**Affiliations:** 1 Dept. of Clinical Sciences, Clinical Research Center, Lund University, Malmö, Sweden; 2 Dept. of Cardiology, Skåne University Hospital, Lund/Malmö, Sweden; 3 Diabetes Center Bioinformatics Unit, Lund University, Malmö, Sweden; 4 Wallenberg Center for Molecular Medicine, Lund University, Malmö, Sweden

**Keywords:** Carotid stenosis, Atherosclerosis, Oxidized low-density lipoproteins, Diabetes mellitus

## Abstract

**Background:**

Type 2 diabetes (T2D) patients are at a greater risk of cardiovascular events due to aggravated atherosclerosis. Oxidized LDL (oxLDL) has been shown to be increased in T2D plaques and suggested to contribute to plaque ruptures. Despite intensified statin treatment during the last decade the higher risk for events remains. Here, we explored if intensified statin treatment was associated with reduced oxLDL in T2D plaques and if oxLDL predicts cardiovascular events, to elucidate whether further plaque oxLDL reduction would be a promising therapeutic target.

**Methods:**

Carotid plaque OxLDL levels and plasma lipoproteins were assessed in 200 patients. Plaque oxLDL was located by immunohistochemistry. Plaque cytokines, cells and scavenger receptor gene expression were quantified by Luminex, immunohistochemistry and RNA sequencing, respectively. Clinical information and events during follow-up were obtained from national registers.

**Results:**

Plaque oxLDL levels correlated with markers of inflammatory activity, endothelial activation and plasma LDL cholesterol (r = 0.22-0.32 and p ≤ 0.01 for all). T2D individuals exhibited lower plaque levels of oxLDL, sLOX-1(a marker of endothelial activation) and plasma LDL cholesterol (p = 0.001, p = 0.006 and p = 0.009). No increased gene expression of scavenger receptors was identified in T2D plaques. The lower oxLDL content in T2D plaques was associated with a greater statin usage (p = 0.026). Supporting this, a linear regression model showed that statin treatment was the factor with the strongest association to plaque oxLDL and plasma LDL cholesterol (p < 0.001 for both). However, patients with T2D more frequently suffered from symptoms and yet plaque levels of oxLDL did not predict cardiovascular events in T2D (findings are summarized in Fig. 1a).

**Conclusions:**

This study points out the importance of statin treatment in affecting plaque biology in T2D. It also implies that other biological components, beyond oxLDL, need to be identified and targeted to further reduce the risk of events among T2D patients receiving statin treatment.

## Introduction

Individuals with type 2 diabetes (T2D) have a markedly increased risk of cardiovascular events and death due to atherosclerosis [1]. Plaque rupture with subsequent atherothrombosis is the major cause of atherosclerotic cardiovascular events in T2D. Longer diabetes duration with increased HbA1c levels has been linked to the formation of rupture prone, so called vulnerable plaques, in T2D patients presenting with acute myocardial infarction [2]. The risk of suffering from a plaque rupture in T2D is likely influenced by several factors including microRNAs, proteolysis of basement membrane, arterial calcification and the intrinsic calcification angle but also more well described plaque components such as the lipid core and a thin fibrous cap [3–6].

Like hyperglycaemia, deregulated lipoproteins are a common condition in T2D and a primary risk factor for cardiovascular disease. The role of modified lipoproteins has been extensively studied in atherosclerosis [7]. Interestingly, triglyceride-rich lipoproteins (TGRL) have demonstrated the ability to increase atherothrombotic risk in fasting T2D patients by means of promoting platelet aggregation via arachidonic acid signalling pathway [8]. Among various modified lipoproteins, oxidized low density lipoprotein (oxLDL) has gained more attention due to its suggested important role in atherosclerotic cardiovascular disease. oxLDL is considered to be crucial for the early plaque formation, to induce inflammatory activity, and is a commonly recognized corner stone of the rupture-prone, (vulnerable plaque phenotype) [9–11]. Previous studies on atherosclerotic plaque tissues collected before 2005, have shown that T2D patients have larger plaques, more frequent plaque ruptures, larger cores of lipids and dead cells, as well as more oxLDL present in their plaques [12–14]. Additionally, circulating levels of oxLDL have been suggested to predict future cardiovascular events in T2D [15].

In 2004, the Collaborative Atorvastatin Diabetes Study (CARDS) showed the beneficial effects of statin treatment among individuals with T2D in preventing future cardiovascular complications [16]. Consequently, the preventive use of statins has increased in T2D patients since then. Although the risk of cardiovascular disease in individuals with T2D has decreased over the last two decades, a clear risk reduction is noticed in the general population as well. This suggests that additional diabetes-specific disease mechanisms responsible for cardiovascular complications remain to be targeted [17].

In this study, we aimed to investigate the effect of intensified statin treatment on oxLDL levels in the plaques of T2D individuals included since 2005. In addition, we wanted to determine if plaque levels of oxLDL predict future cardiovascular events to comprehend if further oxLDL reduction would be a promising therapeutic target in T2D.

## Methods and materials

A detailed description of methods and materials is available as supplementary data (Additional file 1).

### Study Cohort

Carotid plaques and plasma from 200 patients included in the Carotid Plaque Imaging Project (CPIP) biobank were studied. The indications for surgery were 1) plaques with ipsilateral symptoms (stroke, transitory ischemic attack, or amaurosis fugax) and a duplex ultrasound-verified degree of stenosis greater than > 70% or 2) asymptomatic plaques with a degree of stenosis greater than 80%. All patients included were preoperatively assessed by a neurologist. Two patients underwent carotid endarterectomy on two occasions. Informed consent was given by each patient. The study follows the declaration of Helsinki and was approved by the local ethical committee at Lund University.

Plaques were directly snap frozen upon surgical removal. As previously described, a fragment (1 mm) from the most stenotic region of the carotid plaque was kept for histological analysis whereas the rest of the tissue was homogenized [18].

### Clinical information and blood samples

Clinical data regarding risk factors and medications were recorded at the time of inclusion. Among the included patients four different types of statin treatments were recorded, which should have been initiated > 1 week prior to surgery. Statin treatments were divided into low, intermediate or high dose accordingly: Simvastatin (10 mg, 20–30 mg, 40 mg), Pravastatin (10 mg, 20 mg, 40 mg), Atorvastatin (10–30 mg, 40 mg, 80 mg) and Rosuvastatin (5-10 mg, > 10–20 mg, > 20–40 mg).

All blood samples were collected the day before surgery. Plasma levels of low-density lipoprotein cholesterol (LDL), high density lipoproteins (HDL), triglycerides (TG) glycosylated hemoglobin (HbA1c) were all measured using standard procedures. The plasma LDL cholesterol levels were calculated using Friedewald´s equation.

### Assays performed on carotid plaque tissue homogenates

oxLDL levels were measured in the plaque homogenate using ELISA (Mercodia, Uppsala, Sweden). Plaque levels of cytokines, interferon-γ (IFNγ); monocyte chemoattractant protein-1 (MCP-1); macrophage inflammatory protein-1ß (MIP-1ß) and tumor necrosis factor-α (TNF- α) were measured according to the manufacturer’s instructions (human cytokine/chemokine immunoassay; Millipore Corporation, MA), and analysed with Luminex 100 IS 2.3 (Austin, TX). sLOX-1 levels in plaque were measured by the Olink Proseek Multiplex CVD96 × 96 kit (Science for Life Laboratory, Uppsala) and presented in arbitrary units (au).

### Histological analyses carotid plaque tissue sections

Plaques were further phenotyped by histological and immunohistochemical features. Cryosections (8 µm) from the most stenotic part of the plaque were stained for neutral lipids (Oil Red O); macrophages (CD68); smooth muscle cells (α-actin) and oxLDL. Positively stained plaque areas were scanned with Aperio image scope v.8.0 (Aperio, Vista California, USA), and then blindly quantified using Biopix iQ 2.1.8 (Gothenburg, Sweden).

### Carotid plaque RNA-sequencing

RNAseq data of 63 carotid plaques (17 T2D) were used to quantify the expression of the 27 scavenger receptor genes involved in oxLDL uptake. Transcript expression was quantified using Salmon, and DEseq 2 to determine the change in expression levels between patients with and without T2D. Wald test was used to calculate p-values and Benjamin-Hochberg to adjust p-values.

### Clinical follow up

The predictive role of plaque oxLDL levels for future cardiovascular events was explored by clinical follow-up data from the CPIP cohort, as described earlier [19]. Data regarding the clinical follow up was obtained through the Swedish national inpatient health register from October 2005 until December 2015 [19]. All cardiovascular events were identified by hospital discharge codes from the Swedish National Patient Register (with 99% of all somatic and psychiatric hospital discharges registered) and the cardiovascular deaths were obtained from the Swedish Cause of Death Register. Cardiovascular events including death were registered based on the following ICD-10 codes: G45.3, G45.9, G46, I13.2, I209, I21-22, I24.8-9, I25.1-2, I25.5-6, I25.8, I50.9, I60.9, I61.9, I63.1-5, I63.8-9, I64, I64.5, I69.4 and I74.9.

Events were verified by telephone interviews and medical charts. All events within 72 h post carotid endarterectomy were excluded as being procedure-related. For patients suffering from multiple events only the first one was taken into account in the survival analysis.

### Statistics

OxLDL and LDL levels were non-normally distributed. Variables are presented as median and inter quartile range (IQR). Mann–Whitney test (continuous data) and χ2 test (categorical data) were used for two group comparisons. Spearman’s rho was used for correlation analysis. Linear regression was used to determine the effect of statin treatment on plaque oxLDL levels and plasma LDL levels. Survival analysis was performed using Kaplan–Meier curves and Log-rank test. SPSS 22.0 (IBM Corp., Amonk, NY, USA) and GraphPad Prism 8 was used for statistical analysis.

## Results

### Clinical characteristics

The clinical characteristics are presented in Table 1. In general, patients with T2D had lower circulating LDL and total cholesterol, higher body mass index as well as higher triglyceride levels.

**Table 1:** Clinical characteristics of the cohort (n = 200)

	All	No T2D (n = 129)	T2D (n = 71)
Age (years)	69.3 (SD 8.6)	69 (SD 8.8)	70 (SD 8.3)
Sex–Males (%)	134 (67%)	86 (67%)	48 (68%)
Smoking- current/non smokers (%)	65/40 (33/20)	44/24 (34/19)	21/16 (30/23)
BMI^a^	27 (SD 3.9)	26 (SD 3.7)	28 (SD 3.9)***
Degree of stenosis (%)	90 (IQR 80-95)	90 (IQR 80-95)	90 (IQR 75-95)
Hypertension (%)	147 (74)	94 (73)	53 (75)
hsCRP (mg/L)^b^	3.9 (IQR 2.0-6.6)	3.8 (IQR 2-6.7)	4.2 (IQR 1.9-6.6)
HbA1c (mmol/mol)^e^	44 (IQR 38-56)	39 (IQR 36-41)	56 (IQR 47-66)***
Plasma lipoproteins (mmol/L)			
Total cholesterol	4.4 (SD 1.1)	4.6 (SD 1.1)	4.2 (SD 1.1)*
LDL^c^	2.5 (IQR 1.9-3.2)	2.6 (IQR 2.0-3.3)	2.2 (IQR 1.6-3.0)**
HDL^d^	1.1 (IQR 0.9-1.3)	1.1 (IQR 0.9-1.3)	1.0 (IQR 0.8-1.3)
Triglycerides	1.3 (IQR 1.0-1.8)	1.2 (IQR 0.9-1.7)	1.6 (IQR 1.0-2.1)**
Blood glucose lowering treatment, n(%)			
Life style changes	11 (6%)	–	11 (15%)
Oral glucose lowering treatment	37 (19%)	–	37 (52%)
Insulin	22 (11%)	–	22 (31%)
Insulin and oral glucose lowering	12 (6%)	–	12 (17%)
Blood pressure lowering treatment, n(%)			
RAAS inhibitor	101 (51%)	58 (45%)	43 (61%)*
Beta blocker	98 (49%)	59 (46%)	39 (55%)
Statin treatment, n(%)	164 (82%)	100 (78%)	64 (90%)*

Categorical variables are expressed in total amount and percentages. Continuous variables as median and interquartile range (IQR) or mean and standard deviation (SD)

^a^*BMI* Body mass index

^b^*hsCRP* high sensitive CRP

^c^*LDL* Low-density lipoprotein cholesterol

^d^*HDL* High-density lipoprotein

^e^ *HbA1c* hemoglobin A1c, was available for 62% (n = 124) of the cohort. Hypertension defined as: anti-hypertensive treatment or systolic pressure > 140 mmHg. Level of significance between no diabetes and T2D patients is marked by *p < 0.05, **p < 0.01 and ***p < 0.005

### OxLDL is associated with plaque inflammation

**Fig. 1:**
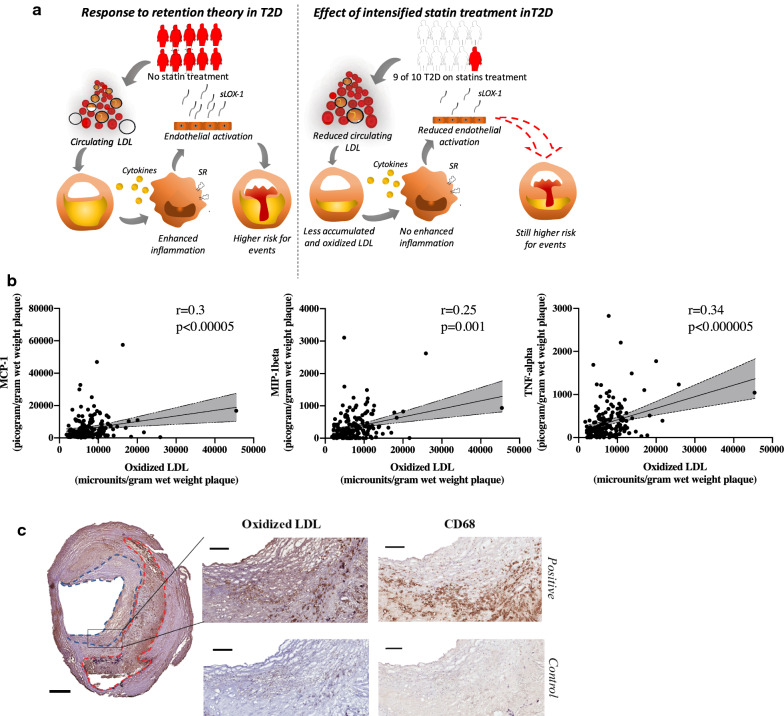
**a** Graphical abstract visualizing the response to retention theory for lipoprotein associated plaque formation and how intensified statin treatment in T2D has affected plaque composition. Individuals marked in red symbolise patients without statin treatment and individuals marked in white symbolise patients receiving statin treatment. LDL, Low density lipoproteins. sLOX-1, soluble LOX-1. SR, scavenger receptors. **b** Plaque levels of oxidized LDL (oxLDL) correlated to plaque levels of the cytokines monocyte chemoattractant protein-1 (MCP-1), macrophage inflammatory protein-1ß (MIP-1ß), and tumour necrosis factor-α (TNF-α). **c** Plaque oxLDL was commonly located in the fibrous cap and the core areas and co-localised with CD68 (both stained dark brown). Scale bars 800 µm and in the magnified area 200 µm. Fibrous cap marked in blue dotted line and core in red dotted line

The effects of oxLDL on a cellular level have been widely studied and suggested to contribute to plaque formation through the induction of inflammation. Herein, plaque levels of inflammatory cytokines (fractalkine, IFN-γ, MIP-1ß, MCP-1 and TNF-α) were measured and correlated with plaque oxLDL levels. When analysing all plaques, oxLDL levels correlated to plaque levels of MCP-1, MIP-1ß and TNF-α (r = 0.3, p < 0.00005; r = 0.25, p = 0.001 and r = 0.34, p < 0.000005, respectively, Fig. 1b). To determine if these correlations were stronger among the group with T2D we analysed plaques from patients with or without T2D separately. However, the same patterns of correlations were identified in both groups (r-range 0.22-0.36) and no significant differences in plaque levels of cytokines were identified when comparing patients with or without diabetes, as previously published [20].

Using immunohistochemistry, we next investigated if oxLDL levels correlated to the macrophage marker CD68, but no significant correlation was identified (r = 0.11, p = 0.1). When dividing the group into plaques from patients with or without T2D, CD68 only correlated to oxLDL levels (r = 0.2, p = 0.03) in plaques from patients without diabetes. Yet, oxLDL and CD68 did co-localise in the plaque (Fig. 1c). Plaque oxLDL levels did not correlate with the plaque area of smooth muscle α-actin. Finally, we identified that oxLDL was commonly located in the core and cap regions of the plaque (Fig. 1c). In summary, these findings confirm previous studies suggesting that oxLDL is associated with plaque inflammatory activity and that no such activity is identified in plaques from patients with T2D.

### Plaque oxLDL levels are reduced in T2D

**Fig. 2:**
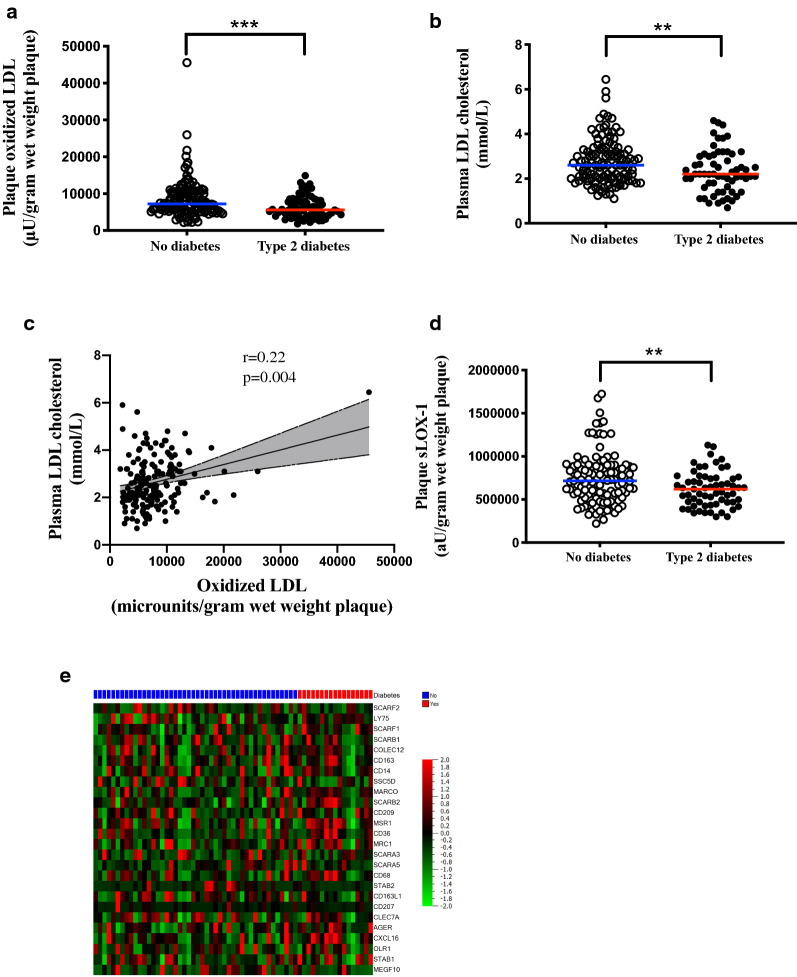
**a** Plaque levels of oxidized LDL (oxLDL) and **b** plasma levels of low density lipoproteins (LDL) cholesterol are reduced in patients with type 2 diabetes (T2D). **c** Plaque levels of oxLDL correlate with circulating LDL cholesterol and **d** plaque levels of soluble LOX-1 (sLOX-1) are reduced in patients with type 2 diabetes (T2D). **e** Heatmap showing no difference in scavenger receptors gene expression levels comparing patients with and without T2D (n = 63). Blue indicates no diabetes and red indicates T2D. Gene expression is mean centred and scaled to unit variance. Colour key indicates increased (red) and decreased (green) intensity associated with normalized expression values

Interestingly, oxLDL levels were found to be significantly lower (22.3%) in plaque tissue from patients with T2D compared to patients without diabetes (5605 (4092–8524) vs 7218 (5538–10485) µU/gram wet weight plaque, p = 0.001; Fig. 2a). In agreement, lower plasma levels of circulating LDL were seen in patients with T2D (2.2 (1.6–3.0) vs 2.6 (2.0–3.3) mmol/L, p = 0.009, Fig. 2b). Plaque levels of oxLDL correlated to plasma levels of total cholesterol (r = 0.2, p = 0.008) and LDL (r = 0.22, p = 0.004, Fig. 2c), whereas no correlations were found with HDL or triglycerides. As lipoprotein modifications have also been suggested to occur under hyperglycaemic conditions we investigated if plaque oxLDL correlated to plasma HbA1c levels. Surprisingly, a trend towards an inverse correlation was detected between plaque oxLDL levels and plasma HbA1c levels (r = −0.17, p = 0.06). In summary, plaque oxLDL and circulating LDL levels are reduced in patients with T2D.

### Associations between plaque oxLDL and markers of cellular oxLDL activation

sLOX-1 is the soluble form of LOX-1, the main oxLDL receptor on endothelial cells, which is released from endothelial cells upon oxLDL stimuli [21].

In line with the reduced levels of oxLDL in T2D, sLOX-1 levels were lower in plaques from patients with T2D compared to plaques from patients without diabetes (620132 (467635–750793) vs 715188 (556317–884383) au/gram wet weight plaque, p = 0.006, Fig. 2d). OxLDL levels correlated with sLOX-1 levels in plaques from both patients with T2D and patients without diabetes (r = 0.32, p = 0.01 and r = 0.28, p = 0.002, respectively). In summary, in support of the reduced oxLDL levels in plaques from patients with T2D, sLOX-1 levels were reduced in plaques from T2D patients.

### Scavenger gene expression is not different in plaques from patients with T2D

The reduced levels of oxLDL identified in plaques from patients with T2D could potentially be explained by an increased cellular uptake and removal of oxLDL. Therefore, plaque gene expression of the main scavenger receptors was assessed in plaque tissue using RNA sequencing. Interestingly, no significant differences were identified (Fig. 2e). In summary, the gene expression of scavenger receptors is not different in plaques from T2D patients compared to patients without diabetes.

### Plaque levels of oxLDL and symptomatic carotid disease

Among patient without diabetes, plasma LDL levels were higher in symptomatic plaques compared to asymptomatic plaques (2.9 (2.2–3.6) vs 2.5 (1.9–3.1) mmol/L, p < 0.05). No such difference was identified in plasma LDL when comparing T2D patients with symptomatic and asymptomatic plaques (2.2 (1.6–3.1) vs 2.2 (1.7–3.0) mmol/L, p = 0.41).

Plaque oxLDL levels were not increased in symptomatic plaques compared to asymptomatic plaques from patients without diabetes (7948 (5934–10889) vs 7169 (5275–9854) µU/gram wet weight plaque, p = 0.09) nor when comparing symptomatic and asymptomatic plaques from patients with T2D (5656 (4333–8178) vs 5431 (3292–8563) µU/gram wet weight plaque, p = 0.5).

The percentage of patients suffering from a symptomatic carotid plaque was significantly higher among T2D patients (65% vs 48%, p = 0.023).

In summary, we show that oxLDL levels are related to circulating LDL, that oxLDL and LDL are not associated with symptoms among patients with T2D and that patients with T2D more frequently suffer from symptomatic carotid plaques.

### Statin treatment is associated with lower LDL and plaque oxLDL levels

**Fig. 3:**
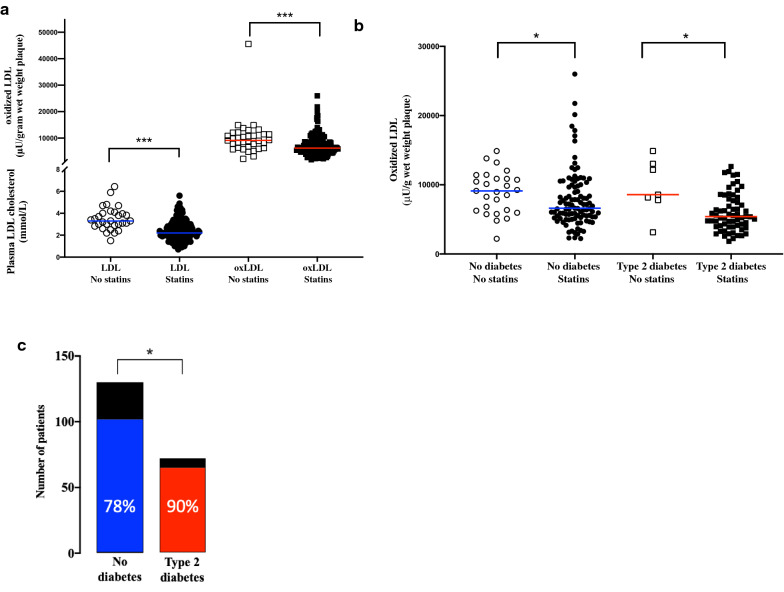
**a** Plasma LDL and plaque oxLDL levels are reduced in patients receiving statin treatment. **b** OxLDL levels are reduced in both patients with and without diabetes with statin treatment compared to patients without statin treatment. **c** The percentage of patients with statin treatment > 1 week prior to surgery was significantly higher in patients with T2D. Blue and red coloured bars indicate the number of patients receiving statin treatment of the all patients in each group (black bars). Percentages of statin treated patients in each group are shown in each bar. Significances are marked by *p < 0.05, ** p < 0.01, ***p < 0.005

As circulating LDL, intima retained LDL and subsequently oxidized LDL are affected by statin treatment we compared if circulating LDL levels and plaque levels of oxLDL were lower in patients with statin treatment. Importantly, both circulating LDL and plaque levels of oxLDL were significantly lower in patients with statin treatment compared to patients without (2.2 (1.8–3.0) vs 3.3 (2.9–4.0) mmol/L, p = 0.0000006 and 6225 (4765–8647) vs 9116 (6352–11435) µU/gram wet weight plaque, p = 0.0002; respectively, Fig. 3a).

When comparing T2D patients with or without statin treatment, even though the group sizes diminished, plaque levels of oxLDL were 37% lower in the group on statin treatment (5405 (3981–7883) vs 8575 (7778–13035) µU/gram wet weight plaque, p = 0.016, Fig. 3b). In the group without diabetes, patients receiving statin treatment had 28% lower oxLDL levels compared to patients without stain treatment (6617 (5324–9893) vs 9186 (6273–11284) µU/gram wet weight plaque, p = 0.017, Fig. 3b). Additionally, as T2D patients received more RAAS inhibitors than patients without diabetes, we further evaluated whether RAAS treatment caused reduction in plaque oxidized LDL. However, no significant difference was identified in plaque oxidized LDL levels when comparing patients with or without RAAS inhibition (6225 (IQR 5005–8938) microunits/gram wet weight plaque vs 7155 (IQR 4252–10512) microunits/gram wet weight plaque; p = 0.2).

Furthermore, the number of patients receiving statin treatment within the week prior to surgery was significantly higher in the T2D group compared to the group without diabetes (64 of 71 patients (90%) with T2D and 100 of 129 patients (78%) without diabetes, p = 0.026, Table 1 and Fig. 3c). The majority of the patients in both groups received Simvastatin treatment, 88% of patients without diabetes and 77% of patients with T2D. The different statins used are summarised in Table 2. Also, 51% of the T2D patients received high dose statin treatment compared to 37% of the patients without diabetes. When oxLDL levels were adjusted for clinical factors including statin treatment, age, gender and T2D diagnosis in a linear regression model, it remained significantly associated to statin treatment (no treatment, low dose, intermediate dose and high dose), T2D diagnosis and age (p = 0.0003, p = 0.014 and p = 0.005, respectively). Also, when LDL levels were adjusted for clinical factors in a linear regression model, statin treatment together with T2D diagnosis remained significantly associated to LDL levels (p = 7x10^−7^ and p = 0.03, respectively). In summary, increased statin treatment was associated with reduced plaque levels of oxLDL in T2D, and T2D patients were more commonly statin treated.

**Table 2:** Summary of different types of statin treatments in type 2 diabetes patients (T2D) and patients without diabetes (No T2D)

	All	No T2D (n = 129)	T2D (n = 71)
Simvastatin, n (high/intermediate/low dose)	137 (76/52/9)	88 (44/37/7)	49 (32/15/2)
Pravastatin, n (high/intermediate/low dose)	7 (4/3/0)	5 (2/3/0)	2 (2/3/0)
Atorvastatin, n (high/intermediate/low dose)	16 (2/4/10)	7 (1/3/3)	9 (1/1/7)
Rosuvastatin, n (high/intermediate/low dose)	4 (1/1/2)	0 (0/0/0)	4 (1/1/2)

Statin treatments were divided into low, intermediate or high dose accordingly: Simvastatin (10 mg, 20–30 mg, 40 mg), Pravastatin (10 mg, 20 mg, 40 mg), Atorvastatin (10–30 mg, 40 mg, 80 mg) and Rosuvastatin (5–10 mg,  > 10–20 mg,  > 20–40 mg)

### Plaque oxLDL levels is not associated to future cardiovascular events

**Fig. 4:**
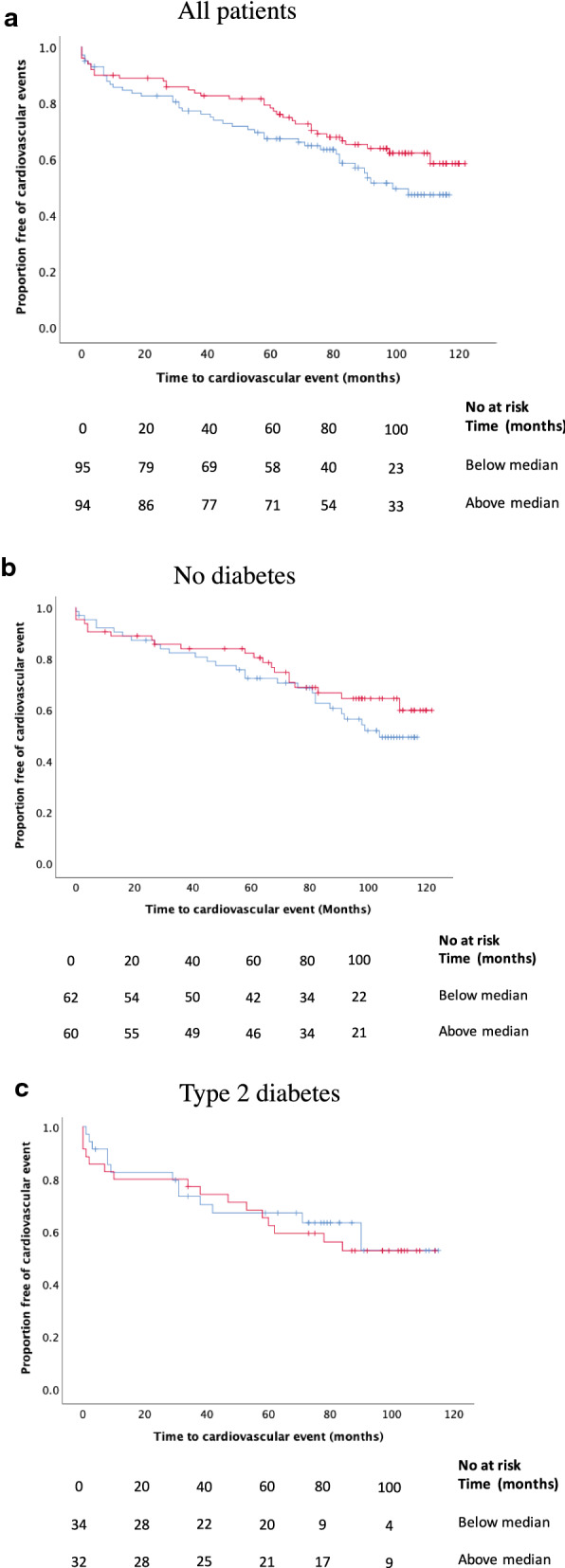
**a** Plaque levels of oxidized LDL(oxLDL) do not predict future cardiovascular events in the whole cohort (p = 0.13) **b**, or in patients without diabetes (p = 0.3) or **c** with T2D (p = 0.76). Red lines indicate plaque levels of oxLDL above median, and blue lines indicate plaque levels of oxLDL below median

oxLDL has been suggested to contribute to the formation of vulnerable plaques, but whether plaque levels of oxLDL do predict cardiovascular events in T2D is unknown. Plaque oxLDL levels were divided into high (above median) and low levels (below median). However, no association between levels of oxLDL and future cardiovascular events was identified in the total cohort (Fig. 4a; Clinical characteristics presented in Additional file 1: Table S1), or when studying the cohort separately in patients with or without diabetes (Fig. 4b, c; Clinical characteristics in Additional file 1: Table SII and SIII). In summary, these findings show that plaque oxLDL does not predict cardiovascular events in a cohort with extensive statin treatment.

## Discussion

Individuals with T2D have previously been shown to exhibit higher plasma oxLDL levels compared to controls [22–25]. Furthermore, atherosclerotic plaques from patients with T2D have also been shown to present larger oxLDL areas, commonly associated with inflammation and a rupture-prone plaque phenotype [13]. Statins are known to reduce plasma lipoproteins (LDL and circulating oxLDL), plaque oxLDL and concurrent inflammation [26, 27]. Atherosclerotic plaques harvested over the past decade show more stable features [28]. Since statins are considered to stabilize the plaques, these changes may be due to increased statin usage [29]. In 2004, the CARDS study reported that treatment of T2D patients with statins reduced their AMI and stroke incidences [16]. Since then, the use of statins has increased in T2D patients. Interestingly, a recent large epidemiological study comparing patients with or without T2D showed a clear reduction in cardiovascular and coronary death between 1998 and 2013. However, the cardiovascular and coronary mortality remains high in T2D and the risk reduction was seen both in T2D and in controls [17]. In line with the reduced frequency of cardiovascular events, recent studies investigating plaque biology have not been able to identify increased inflammatory activity in T2D plaques [20, 30]. This spurred us to explore if the intensified statin treatment affects plaque oxLDL levels in T2D and to evaluate if plaque oxLDL is a predictive marker for future cardiovascular events in T2D. Herein we report that even though increased statin usage in T2D patients reduced their plaque oxLDL levels, the risk for CVD remained comparable with patients without T2D (Fig. 1a).

### oxLDL and carotid plaque inflammation in T2D patients’

In contrast to previous studies demonstrating increased levels of modified LDL including oxLDL in the plasma of T2D patients [31, 32], we found markedly reduced oxLDL levels in the actual plaques from T2D patients. Plaque oxLDL levels have been linked to plaque inflammation and unstable symptomatic carotid plaques [33, 34]. As macrophage infiltration of the fibrous cap is generally seen in symptomatic and/or rupture prone high risk plaques [35], and oxLDL correlates to plaque macrophage content [34], we anticipated that oxLDL levels might reflect the inflammatory status of the plaque. Although oxLDL only correlated to CD68 in plaques from patients without diabetes, it did correlate to pro-inflammatory cytokine levels in both groups. However, inflammatory activity in plaques from patients with T2D did not vary considerably from plaques from patients without diabetes and neither did we observe stronger associations in the T2D group. This finding is in line with a recent large histological study by Scholtes et al. showing that there is no longer any enhanced inflammatory activity to detect in T2D plaques [30].

### Cellular activation and uptake of oxLDL in carotid plaques of T2D patients

Even though the oxLDL content was reduced in T2D plaque tissues, its biological effect in the tissue could still be greater depending on the cellular response. To explore if a more potent biological effect of oxLDL was evident in T2D plaque tissue we measured plaque levels of sLOX-1 (the oxidized LDL receptor), which is a soluble form of LOX-1, an oxLDL receptor expressed by the majority of plaque cells [36, 37]. sLOX-1 is released by proteolytic cleavage of the extracellular domain of LOX-1 [38] and we have demonstrated that sLOX-1 release is induced by oxLDL [21]. Interestingly, along with decreased oxLDL levels, the plaque content of sLOX-1was reduced in T2D plaques suggesting that there is no increased endothelial cell activation due to a greater cellular response to oxLDL in T2D plaques.

An increased uptake of oxLDL could also potentially explain the reduced plaque content of oxLDL in T2D patients. Active uptake of oxLDL through one or more cellular scavenger receptors could be reflected in the increased surface expression of these receptors [39]. Therefore, we assessed the gene expression of scavenger receptors in the plaque tissue to determine if this reduction is caused by increased cellular engulfment of oxLDL in the plaques of T2D patients. Surprisingly, no difference in the expression of any scavenger receptor was detected comparing plaques from patients with or without diabetes. These results suggest that the low oxLDL levels in T2D plaques are not due to its increased cellular uptake or metabolism.

### Effect of statins on plasma LDL and plaque oxLDL levels in T2D patients

Past studies have convincingly showed that T2D patients have no significant increase in the circulating LDL particles compared to controls [40]. However, it is presumed that T2D patients still have more oxLDL as they endure several qualitative changes in LDL particles, such as increased oxidation [41]. In the present study both plaque oxLDL and plasma LDL levels were significantly lower in T2D patients (but only in the plaque of those receiving statins). Furthermore, plasma LDL levels significantly correlated with plaque oxLDL in T2D patients which supports previous studies pointing out the importance of circulating LDL for the retention and oxidation of LDL in the arterial wall.

As statin treatment is known to affect both LDL and oxLDL we aimed to investigate if the reduced LDL and oxLDL levels were associated to increased statin treatment among patients with T2D. Importantly, the frequency of patients receiving statin treatment was significantly higher in the T2D group. Also, a higher percentage of the T2D patients received high dose statin treatment and when adjusted for clinical factors, statin treatment had a significant influence on both oxLDL and LDL levels. This implies that the reduced LDL and oxLDL levels observed in the T2D group are likely to be due to a more extensive statin treatment, in line with the clinical guidelines.

### Predictive value of plaque oxLDL for cardiovascular events in T2D patients

Finally, in view of present interest in finding possible predictors for cardiovascular events, we investigated, (to our knowledge for the first time) whether plaque oxLDL levels predict cardiovascular events. In contrast to current reports regarding plasma levels of oxLDL [15], we could not find any association of high plaque levels oxLDL with future CVD events. It could be inferred here that as statins decreased the oxLDLs in plaques of T2D patients, in the course of time, other mechanisms could become relevant to the formation and rupture of the culprit plaque phenotype in T2D patients. Of note, small dense LDL knowingly have synergistic effect on cardiovascular disease especially in higher-risk patients such as T2D [42]. High prevalence of oxidized and small, dense LDL levels is found in T2D patients [43], and they have been demonstrated as a predictor of atherosclerotic plaque progression [44]. Based on the design of the current study, where we aimed to explore if T2D plaque oxLDL levels were reduced due to a greater statin usage, we cannot exclude that plaques from patients with T2D under statin treatment can still be rich in small dense LDL particles.

## Limitations

There are limitations in the current study that need to be considered. First, the cross-sectional design of the study does not allow for establishing a cause-effect relationship between plaque oxLDL levels and statin usage. However, the oxLDL reducing effect of statins has been proven and the aim of the current study was, in addition to previous studies, to explore if the increased statin usage in T2D has changed the biological composition of the plaques. Furthermore, the oxLDL ELISA only assessed one epitope on the oxidized ApoB100 and it cannot be ruled out that other lipid remnants are increased in T2D plaques. However, sLOX-1 as a marker of oxLDL cell activation, (not only reflecting the assessed epitope), was also reduced in T2D plaques. It should also be considered that the study cohort is small in terms of follow up, even though it is a large study considering the human plaque analyses. Thus, considering the fact that no trend towards a higher risk for events with high levels of oxLDL was seen, these findings are of clinical interest.

Furthermore, no information regarding diabetes duration was available to adjust for. However, the durations of diabetes, which is based on the year the patient was diagnosed with diabetes can also be misleading as T2D is often a silent disease until complications appear, patients may have suffered from hyperglycemia for a long period of time before diagnosis.

The identified trend towards an inverse correlation between oxLDL and HbA1c was identified. This finding could potentially be explained by the fact that statin treatment itself has been shown to increase HbA1c levels [45, 46].

Finally, all patients included in the present suffer from advanced carotid atherosclerotic disease. Herein, no conclusions can be drawn regarding the biological effect of statin treatment in patients without an advanced atherosclerotic disease.

## Conclusions

In conclusion, the present study shows that plaque oxLDL levels and circulating LDL levels are reduced in patients with T2D. These biological changes are likely to be the result of a more intensive statin treatment and support the important role of affecting plaque biology with preventive statin treatment in T2D. Yet, the frequency of plaque ruptures was still higher among patients with T2D and plaque oxLDL levels did not predict future cardiovascular events. Taken together these findings suggest that other therapeutic targets, next to lipid lowering strategies, are needed to further reduce the risk for cardiovascular complications among patients with T2D.

## Supplementary information

**Additional file 1.** Supplementary methods and table SI, SII and SIII.

## Data Availability

The datasets generated and/or analysed during the current study are not publicly available due the sensitive nature of the data, requests to access the dataset from qualified researchers trained in human subject confidentiality protocols may be sent to Isabel Goncalves at Lund University.

## Abbreviations

oxLDL: Oxidized LDL
LDL: Low density lipoproteins cholesterol
HbA1C: Hemoglobin A1c
T2D: Type 2 diabetes
CD68: Cluster of differentiation 68
sLOX-1: Soluble lectin-type oxidized LDL receptor 1
CVD: Cardiovascular disease
IFN-γ: Interferon-γ
MIP-1ß: Macrophage inflammatory protein 1ß
MCP-1: Monocyte chemoattractant protein-1
TNF-α: Tumor necrosis factor-α
