# Correction to: Reduced oxidized LDL in T2D plaques is associated with a greater statin usage but not with future cardiovascular events

**DOI:** 10.1186/s12933-021-01227-4

**Published:** 2021-03-12

**Authors:** Pratibha Singh, Isabel Goncalves, Christofer Tengryd, Mihaela Nitulescu, Ana F. Persson, Fong To, Eva Bengtsson, Petr Volkov, Marju Orho-Melander, Jan Nilsson, Andreas Edsfeldt

**Affiliations:** 1 Dept. of Clinical Sciences, Clinical Research Center, Lund University, Malmö, Sweden; 2 Dept. of Cardiology, Skåne University Hospital, Lund/Malmö, Sweden; 3 Diabetes Center Bioinformatics Unit, Lund University, Malmö, Sweden; 4 Wallenberg Center for Molecular Medicine, Lund University, Malmö, Sweden

## Correction to: Cardiovasc Diabetol (2020) 19:214 https://doi.org/10.1186/s12933-020-01189-z

Following publication of the original article [1], the authors provided the additional corrections which were not included by the typesetter before publication. The following corrections are presented with this erratum.

In Introduction section, the beginning sentence of the second paragraph should read as, “Like hyperglycaemia, dysregulated lipoproteins are a common condition in T2D and a primary risk factor for cardiovascular disease”.

The correct subsection title should read as, “Histological analyses of carotid plaque tissue sections”.

In OxLDL is associated with plaque inflammation subsection, the last sentence in the second paragraph should read as, “In summary, these findings confirm previous studies suggesting that oxLDL is associated with plaque inflammatory activity and that no increased inflammatory activity is identified in plaques from patients with T2D”.

In Plaque levels of oxLDL and symptomatic carotid disease subsection, the first sentence should read as, “Among patients without diabetes, plasma LDL levels were higher in symptomatic plaques compared to asymptomatic plaques (2.9 (2.2–3.6) vs 2.5 (1.9–3.1) mmol/L, p < 0.05).”

The corrected captions of Figs. 2 and 3 are given below:

**Figure 2 a** Plaque levels of oxidized LDL (oxLDL) and **b** plasma levels of low density lipoproteins (LDL) cholesterol are reduced in patients with type 2 diabetes (T2D). **c** Plaque levels of oxLDL correlate with circulating LDL cholesterol and **d** plaque levels of soluble LOX-1 (sLOX-1) are reduced in patients with type 2 diabetes (T2D). Blue and red lines indicate the median. **e** Heatmap showing no difference in scavenger receptors gene expression levels comparing patients with and without T2D (n = 63). Blue indicates no diabetes and red indicates T2D. Gene expression is mean centred and scaled to unit variance. Colour key indicates increased (red) and decreased (green) intensity associated with normalized expression values.

**Figure 3 a** Plasma LDL and plaque oxLDL levels are reduced in patients receiving statin treatment. **b** OxLDL levels are reduced in both patients with type 2 diabetes (T2D) and without diabetes with statin treatment compared to patients without statin treatment. **c** The percentage of patients with statin treatment > 1 week prior to surgery was significantly higher in patients with T2D. Blue and red coloured bars indicate the number of patients receiving statin treatment of all patients in each group (black bars). Percentages of statin treated patients in each group are shown in each bar. Significances are marked by *p < 0.05, ** p < 0.01, ***p < 0.005.

In subsection, “Statin treatment is associated with lower LDL and plaque oxLDL levels”, the beginning sentences of third paragraph should read as, “Furthermore, the number of patients receiving statin treatment more than 1 week prior to surgery was significantly higher in the T2D group compared to the group without diabetes (64 of 71 patients (90%) with T2D and 100 of 129 patients (78%) without diabetes, p = 0.026, Table 1 and Fig. 3c). The majority of the statin treated patients in both groups received Simvastatin treatment, 88% of patients without diabetes and 77% of patients with T2D. The different statins used are summarised in Table 2”.

In the first section of the Discussion, the last sentence should read as, “*Herein, we report that even though increased statin usage among T2D patients has reduced plaque oxLDL levels, the risk to have suffered from a symptomatic carotid plaque remained significantly higher in the T2D group* (Fig. 1a)”.

**Fig. 1:**
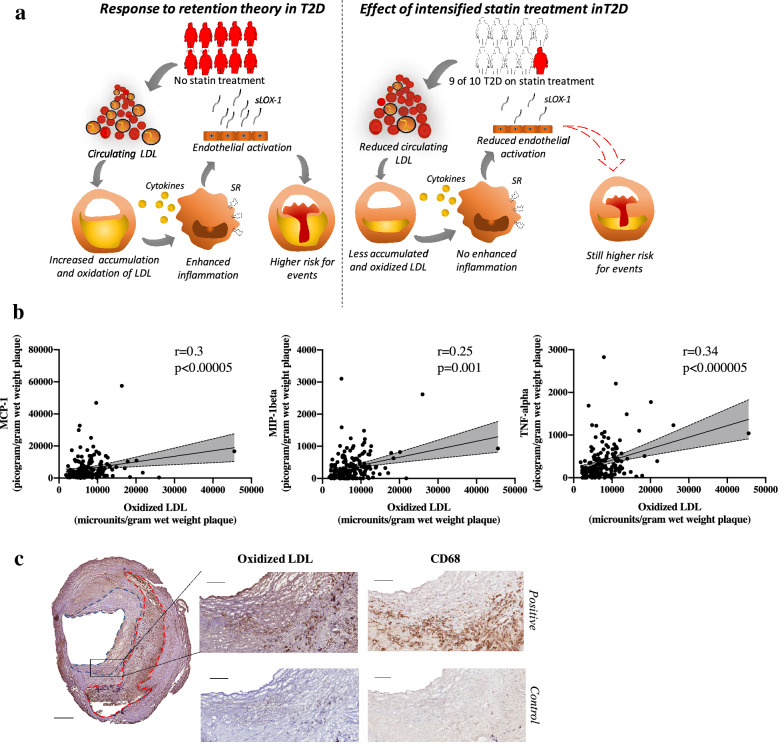
**a** Graphical abstract visualizing the response to retention theory for lipoprotein associated plaque formation and how intensified statin treatment in type 2 diabetes (T2D) has affected plaque composition. Individuals marked in red symbolise patients without statin treatment and individuals marked in white symbolise patients receiving statin treatment. LDL, Low density lipoproteins. sLOX-1, soluble LOX-1. SR, scavenger receptors.** b** Plaque levels of oxidized LDL (oxLDL) correlated to plaque levels of the cytokines monocyte chemoattractant protein-1 (MCP-1), macrophage inflammatory protein-1ß (MIP-1ß), and tumour necrosis factor-α (TNF-α).** c** Plaque oxLDL was commonly located in the fibrous cap and the core areas and co-localised with CD68 (both stained dark brown). Scale bars 800 µm and in the magnified area 200 µm. Fibrous cap marked in blue dotted line and core in red dotted line

In Limitations section, the first sentence of third paragraph should read as, “We also identified a trend towards an inverse correlation between oxLDL and HbA1c”.

The corrected Abbreviations are given below:

sLOX-1: Soluble lectin-type oxidized LDL receptor-1; MIP-1ß: Macrophage inflammatory protein-1ß.

In Funding section, “Åke Wiberg foundation” should be included.

The original article [1] has been updated.
