# Supplementary material for: Reduced oxidized LDL in T2D plaques is associated with a greater statin usage but not with future cardiovascular events
Source: Cardiovasc Diabetol. 2020 Dec 14;19:214. doi: 10.1186/s12933-020-01189-z (PMC7737372; doi:10.1186/s12933-020-01189-z)
Supplement: Supplementary file 1 — Additional file 1. Supplementary methods and table SI, SII and SIII. [file 12933_2020_1189_MOESM1_ESM.docx]

**SUPPLEMENTARY METHODS AND TABLES**

**“Reduced oxidized LDL plaque levels in type 2 diabetes is associated to a greater statin usage and not associated to future cardiovascular events”**

***Research Design and Methods***

Carotid plaques and plasma from 200 patients included in the Carotid Plaque Imaging Project (CPIP) biobank were studied. The indications for surgery were 1) plaques with ipsilateral symptoms (stroke, transitory ischemic attack, or amaurosis fugax) and a duplex ultrasound-verified degree of stenosis greater than >70% or 2) asymptomatic plaques with a degree of stenosis greater than 80%. All patients included were preoperatively assessed by a neurologist. Clinical data regarding risk factors were recorded at the time of inclusion.

The four different types of statin treatment used were divided into low, intermediate and high dose treatment accordingly: Simvastatin (10mg, 20-30mg, 40mg), Pravastatin (10mg, 20mg, 40mg), Atorvastatin (10-30mg, 40mg, 80mg) and Rosuvastatin (5-10mg, >10-20mg, >20-40mg).

Plasma was collected the day before surgery. Two patients underwent carotid endarterectomy at two occasions. Informed consent was given by each patient. The study follows the declaration of Helsinki and was approved by the local ethical committee at Lund University.

Plaques were directly snap frozen upon surgical removal. As previously described, a fragment (1 mm) from the most stenotic region of the carotid plaque was kept for histological analysis whereas the rest of the tissue was homogenized ^1^.

*Oxidized LDL and cytokines in plaque tissue homogenates*

Plaque homogenate supernatant levels of oxLDL were assessed using ELISA (Mercodia, Uppsala, Sweden). The analysis was performed in accordance to the manufacturer´s instructions. Supernatants were obtained by centrifugation of plaque homogenate at 13000 g for 10 minutes for cytokine analysis and 12000g for oxLDL measurement. The pro-inflammatory cytokines interferon-γ (IFNγ), monocyte chemoattractant protein-1 (MCP-1), macrophage inflammatory protein-1ß (MIP-1ß) and tumor necrosis factor-**α (TNF- α) were quantified in plaque tissue homogenate supernatants using a** human cytokine/chemokine immunoassay (Millipore Corporation, MA) and analysed with Luminex 100 IS 2.3 (Austin, Texas, USA), according to the manufacturer´s instructions.

*Assessment of plaque soluble lectin-like oxidized low-density lipoprotein receptor-1 (sLOX-1) levels*

Plaque levels of sLOX-1 levels were measured in plaque tissue homogenates in a subgroup of 186 plaques using the Olink Proseek Multiplex CVD^96x96^ kit (Clinical Biomarkers Facility, Science for Life Laboratory, Uppsala University). Plaque levels of sLOX-1 are presented in arbitrary units (au) on a log2 scale. Lower and upper limits of quantification correspond to 1.0 and 15625 pg/mL (www.olink.com). The within- and between-run coefficients of variation were 9% and 11%.

*Histological and immunohistochemically analyses*

The 1 mm segment from the most stenotic part of the plaque was cryo-sectioned into 8 µm sections. Sections were then stained for neutral lipids (Oil Red O), macrophages (CD68) and smooth muscle cells (α-actin) as previously described ^2^. When staining for oxLDL, a primary mouse–human chimera of 2D03 anti-ox-LDL antibody (mouse IgG2a LDO-2D03) with murine constant regions and human variable regions was used (Bioinvent- PID 3603/050526 ZA) and a secondary biotinylated rabbit anti-mouse F(ab’)2 (E0413; DakoCytomation) were used. An isotype control antibody, mouse IgG2a FITC-8 was used as a control (Bioinvent). Positively stained plaque areas were scanned and photographed with Aperio image scope v.8.0 (Aperio, Vista Californien, USA), and then blindly quantified using Biopix iQ 2.1.8 (Gothenburg, Sweden).

*RNA sequencing*

mRNA expression of the 27 scavenger receptor genes (downloaded from HGNC under gene group: scavenger receptor) was evaluated from global transcriptome RNAseq data of 63 carotid plaques: (17 with type 2 diabetes). RNA was prepared from standard total RNA extraction with Trizol cleared of Ribosomal RNA using Ribo-Zero™ Magnetic Kit from (Epicentre). Strand specific RNAseq libraries were prepared with ScriptSeq™ v2 RNA-Seq Library v2 Preparation Kit (Epicentre). Paired-end sequencing libraries for 63 RNA samples were generated and sequenced using high-output kit version 2, HiSeq2000 platform, Illumina, USA. Adapter sequences and bases with Phred score less than 20 were removed from the sequencing reads using TrimGalore! (Version 0.3.7, Babraham Bioinformatics, Cambridge, UK) and quality controls were performed using FastQC (Version 0.11.2, Babraham Bioinformatics, Cambridge, UK) and summarized using mutliqc (Version 0.9). Transcript expression of all 63 samples was quantified using Salmon ^3^ with transcript annotations from Gencode v.27, and, subsequently, gene-level counts were summarized using tximport ^4^. Next, DEseq2 differential expression analysis ^5^ was used to determine the change in expression level of scavenger receptor genes between plaques from patients with or without type 2 diabetes and Wald test was used to calculate p-values. Benjamin-Hochberg corrections were used to calculate adjusted p-values after correcting for multiple testing. Heatmap of expression data was plotted using mean centered counts normalized to unit variance in Qlucore omics explorer v 3.5.

*Clinical follow up*

The predictive role of plaque oxLDL levels for future cardiovascular events was explored by clinical follow-up from the CPIP cohort, as described ^6^. Data regarding the clinical follow up was obtained through the nationwide Swedish national inpatient health register from October 2005 until December 2015^6^. All cardiovascular events were identified by hospital discharge codes from the Swedish National Patient Register (with 99% of all somatic and psychiatric hospital discharges registered) and the cardiovascular deaths were obtained from the Swedish Cause of Death Register. Cardiovascular events including cardiovascular death were registered based on the following ICD-10 codes: G45.3, G45.9, G46, I13.2, I209, I21-22, I24.8-9, I25.1-2, I25.5-6, I25.8, I50.9, I60.9, I61.9, I63.1-5, I63.8-9, I64, I64.5, I69.4 and I74.9.

Events were verified by telephone interviews and medical charts. All events within 72 hours post carotid endarterectomy were excluded as being procedure-related. For patients suffering of multiple events only the first one was taken into account in the survival analysis.

*Statistics*

OxLDL and LDL levels were non-normally distributed. Variables are presented as median and inter quartile range (IQR). Mann-Whitney test (continuous data) and χ2 test (categorical data) were used for two group comparisons. Spearman’s rho was used for correlation analysis. Linear regression was used to determine the effect of statin treatment on plaque oxLDL levels and plasma LDL levels. Survival analysis was performed using Kaplan-Meier curves and Log-rank test. SPSS 22.0 (IBM Corp., Amonk, NY, USA) and GraphPad Prism 7 were used for statistical analysis.

**Supplemental Table SI.** *Clinical characteristics of the study cohort divided by the median levels of plaque oxidized LDL (n=196).*

|  | Below median  (n=98) | Above median  (n=98) |
| --- | --- | --- |
| Age (years) | 72 (67-77) | 68 (63-74)*** |
| Gender – Males (%) | 66 (67%) | 66 (67%) |
| Smoking- current/nonsmokers(%) | 22/25 (22/26) | 42/13 (43/13)*** |
| BMI* | 26.4 (SD 3.7) | 26.8 (SD 3.9) |
| Degree of stenosis (%) | 90 (80-95) | 90 (80-95) |
| Hypertension | 75 (77) | 68 (69) |
| Statin treatment (%) | 90 (92) | 72 (73)*** |
| hsCRP (mg/L) † | 4 (2.0-6.7) | 3.9 (2-6.6) |
| Cholesterol (mmol/L) | 4.3(SD 1) | 4.5(SD1) |
| LDL (mmol/L) ‡ | 2.2 (1.8-3.0) | 2.6 (2-3.3) * |
| HDL (mmol/L) § | 1.2 (0.9-1.4) | 1.0 (0.9-1.2) |
| Triglycerides (mmol/L) | 1.2 (0.9-1.8) | 1.4 (1.0-1.9) |
| HbA1c (mmol/mol) \|\| | 45.8 (39-59) | 42 (37-54) |

Categorical variables are expressed as numbers and percentages. Continuous variables are expressed as median and interquartile range (IQR) or mean and standard deviation (SD). *BMI, Body mass index. †hsCRP, high sensitive C-reactive protein. ‡LDL, Low-density lipoprotein. §HDL, High-density lipoprotein. || HbA1c, hemoglobin A1c, was available for 61% (n=120) of the included patients. Hypertension is defined as anti-hypertensive treatment or systolic pressure > 140 mmHg.

Level of significance is marked by *p<0.05, **p<0.01 and ***p<0.005.

**Supplemental Table SII.** *Clinical characteristics of patients without diabetes divided by the median levels of plaque oxidized LDL (n=126).*

|  | Below median  (n=63) | Above median  (n=63) |
| --- | --- | --- |
| Age (years) | 70 (SD 7.6) | 67 (SD 9.8)* |
| Gender – Males (%) | 43 (67) | 42 (66) |
| Smoking- current/nonsmokers(%) | 17/14 (27/22) | 26/9 (41/14) |
| BMI* | 26 (24-27) | 27 (24-29) |
| Degree of stenosis (%) | 90 (80-95) | 90 (80-95) |
| Hypertension (%) | 48 (76) | 43 (68) |
| Lipid-lowering treatment (%) | 55 (87) | 44 (70)* |
| hsCRP (mg/L) † | 4.1 (2.0-6.8) | 3.2 (2-6.8) |
| Cholesterol (mmol/L) | 4.3 (3.5-5.3) | 4.6 (3.9-5.2) |
| LDL (mmol/L) ‡ | 2.4 (1.9-3.1) | 3.0 (2.1-3.4) |
| HDL (mmol/L) § | 1.2 (0.9-1.4) | 1.1 (0.9-1.2) |
| Triglycerides (mmol/L) | 1.2 (0.8-1.5) | 1.3 (0.9-1.8) |

Categorical variables are expressed as numbers and percentages. Continuous variables are expressed as median and interquartile range (IQR) or mean and standard deviation (SD). *BMI, Body mass index. †hsCRP, high sensitive C-reactive protein. ‡LDL, Low-density lipoprotein. §HDL, High-density lipoprotein. Hypertension is defined as anti-hypertensive treatment or systolic pressure > 140 mmHg.

Level of significance is marked by *p<0.05, **p<0.01 and ***p<0.005.

**Supplemental Table SIII.** *Clinical characteristics of the patients with type 2 diabetes divided by the median levels of plaque oxidized LDL (n=70).*

|  | Below median  (n=35) | Above median  (n=35) |
| --- | --- | --- |
| Age (years) | 71 (66-77) | 69 (61-77) |
| Gender – Males (%) | 22 (%) | 26 (%) |
| Smoking- current/nonsmokers(%) | 8/9 (23/26) | 13/6 (37/17) |
| BMI* | 28 (25-31) | 28 (26-31) |
| Degree of stenosis (%) | 90 (75-95) | 90 (75-95) |
| Hypertension | 26 (74) | 26 (74) |
| Lipid-lowering treatment | 34 (97) | 29 (83)* |
| hsCRP (mg/L) † | 3.3 (1.9-6.6) | 4.7 (2.4-6.7) |
| Cholesterol (mmol/L) | 4.2 (3.2-4.8) | 4.3 (3.5-5.1) |
| LDL (mmol/L) ‡ | 2.2 (1.6-2.6) | 2.3 (1.6-3.1) |
| HDL (mmol/L) § | 1.1 (0.8-1.4) | 1.0 (0.9-1.2) |
| Triglycerides (mmol/L) | 1.5 (1.0-1.8) | 1.6 (1.2-2.4) |

Categorical variables are expressed as numbers and percentages. Continuous variables are expressed as median and interquartile range (IQR) or mean and standard deviation (SD). *BMI, Body mass index. †hsCRP, high sensitive C-reactive protein. ‡LDL, Low-density lipoprotein. §HDL, High-density lipoprotein. Hypertension is defined as anti-hypertensive treatment or systolic pressure > 140 mmHg.

Level of significance is marked by *p<0.05, **p<0.01 and ***p<0.005.

*References*

1. Goncalves I, Moses J, Dias N, Pedro LM, Fernandes e Fernandes J, Nilsson J, Ares MP. Changes related to age and cerebrovascular symptoms in the extracellular matrix of human carotid plaques. Stroke 2003;**34**(3):616-22.

2. Edsfeldt A, Grufman H, Asciutto G, Nitulescu M, Persson A, Nilsson M, Nilsson J, Goncalves I. Circulating cytokines reflect the expression of pro-inflammatory cytokines in atherosclerotic plaques. Atherosclerosis 2015;**241**(2):443-9.

3. Patro R, Duggal G, Love MI, Irizarry RA, Kingsford C. Salmon provides fast and bias-aware quantification of transcript expression. Nat Methods 2017;**14**(4):417-419.

4. Soneson C, Love MI, Robinson MD. Differential analyses for RNA-seq: transcript-level estimates improve gene-level inferences. F1000Res 2015;**4**:1521.

5. Love MI, Huber W, Anders S. Moderated estimation of fold change and dispersion for RNA-seq data with DESeq2. Genome Biol 2014;**15**(12):550.

6. Goncalves I, Singh P, Tengryd C, Cavalera M, Yao Mattisson I, Nitulescu M, Flor Persson A, Volkov P, Engstrom G, Orho-Melander M, Nilsson J, Edsfeldt A. sTRAIL-R2 (Soluble TNF [Tumor Necrosis Factor]-Related Apoptosis-Inducing Ligand Receptor 2) a Marker of Plaque Cell Apoptosis and Cardiovascular Events. Stroke 2019;**50**(8):1989-1996.
